# Serological differentiation between naturally acquired mpox and MVA-BN-vaccine induced antibody responses using ratios of MPXV and VACV antigen pairs in the MSD immunoassay

**DOI:** 10.1128/spectrum.00182-25

**Published:** 2025-08-08

**Authors:** Aleksandra Pettke, Marton Keszei, Wanda Christ, Nuria Mayola Danés, Sara Gredmark-Russ, Sandra Söderholm, Finn Filén, Elisabet Storgärd, Victor Westergren, Victor Yman, Johan Aarum, Jonas Klingström, Kari Johansen, Anna Mia Ekström, Klara Sondén

**Affiliations:** 1 Public Health Agency of Sweden 25545, Solna, Sweden; 2 Department of Clinical Science and Education, Södersjukhuset Karolinska Institutet, Stockholm, Sweden; 3 Department of Microbiology Tumor and Cell Biology, Karolinska Institutet 310388, Stockholm, Sweden; 4 Department of Medicine Huddinge, Centre for Infectious Medicine, Karolinska Institutet, Stockholm, Sweden; 5 Department of Infectious Diseases, Karolinska University Hospital 59562, Stockholm, Sweden; 6 Laboratory for Molecular Infection Medicine Sweden 168538 , Umeå, Sweden; 7 Department of Infectious Diseases/Venhälsan Södersjukhuset, Stockholm, Sweden; 8 Department of Medicine Solna, Karolinska Institutet 27106, Stockholm, Sweden; 9 Department of Biomedical and Clinical Sciences, Linköping University 4566, Linköping, Sweden; 10 Department of Global Public Health, Karolinska Instititutet, Stockholm, Sweden; Naturwissenschaftliches und Medizinisches Institut an der Universitat Tubingen, Reutlingen, Germany

**Keywords:** MPXV, VACV, serology, immunization, differentiation

## Abstract

This study’s aim was to evaluate and benchmark the gold-standard methods neutralization test (NT) and immunofluorescence (IF), against a model linked to a multiplex immunoassay method for analysis of the highly cross-reactive serological responses against the monkeypox (MPXV)- and vaccinia (VACV) viruses. Serum samples from men who have sex with men were analyzed (22 with previous PCR-confirmed mpox episode, 53 MVA-BN-vaccinated, 61 unexposed to infection/vaccination). In-house NTs and IF established titers of mpox- and vaccinia-specific neutralizing and binding IgG antibodies, respectively. The commercial Orthopoxvirus Panel 1 multiplex immunoassay (Meso Scale Discovery [MSD]) was used to measure IgG against five homologous MPXV and VACV antigens. Antibody titers were studied in relation to infection and vaccination. The gold-standard NT and IF assays had ≥90% sensitivity identifying mpox-infected individuals. Despite high specificities in the unexposed group (≥91.8%), serum samples of vaccinated individuals strongly cross-reacted in the gold-standard assays with specificities of 73.6% (MPXV NT) and 43.4% (MPXV IF). Similarly, in the MSD assay, no individual antigen could differentiate between MPXV- or VACV-specific IgG antibodies. However, using ratios of IgG concentrations against three MPXV/VACV antigens enabled classification of separated mpox infected, vaccinated, and unexposed individuals with 88.9% accuracy. Through an innovative analytic approach, utilizing antibody response ratios to homologous MPXV and VACV antigen pairs, we demonstrate a model which differentiates MPXV-infected individuals from vaccinated and unexposed. This approach shows promise as a tool for transmission surveillance and outbreak monitoring, particularly in resource poor settings with low diagnostic capacity, and to prioritize vaccine.

**IMPORTANCE:**

Our study provides a uniquely broad perspective on orthopox serology by contrasting the gold standard NT and IF methods with the novel MSD multiplex assay and by providing an analysis model of multiplex data to separate previously mpox-infected, MVA-BN-vaccinated, and unexposed individuals.

## INTRODUCTION

The WHO declaration of the monkeypox virus (MPXV) outbreaks as a public health emergency of international concern (PHEIC) in August 2024 (1). This highlighted the urgent need for reliable diagnostic tests and for advancing and refining serological methods to monitor incidence and prevalence of existing and evolving immunity in populations at high risk.

MPXV, a double-stranded DNA virus in the orthopoxvirus (OPXV) family, is characterized by high antigenic similarity across the viral strains. The cross-immunity between OPXVs has been exploited using the live replication-deficient modified Vaccinia Virus Ankara Bavarian Nordic A/S, (MVA-BN) which initially was developed and approved against smallpox but also provides significant protection against the closely related MPXV (clade 2b) (2, 3).

Serological assays play a key role in facilitating seroepidemiological surveillance, detecting previous asymptomatic infections and delineating immune responses to both natural infection and vaccination. However, there is a lack of affordable, commercially available serology assays which can be performed without the need of high containment laboratories. In-house developed, gold-standard methods based on live viruses like neutralization assays (NT) or immunofluorescence assays (IFA) are work-intensive and require high containment facilities (4).

In light of global vaccine shortages and the growing mpox outbreak in Africa (5), testing for immunity before vaccination may be a way forward to save vaccine doses. However, a high level of sequence homology between OPXV antigenic epitopes (6) complicates the differentiation of previously mpox-infected and -MVA-BN-vaccinated individuals. A number of in-house and commercial assays were developed to circumvent this methodological bottleneck. MPXV B21R peptides have been described to identify mpox caused by several different strains of the virus (7, 8). Moreover, a study by Otter et al. has identified MPXV A27 as a serological marker of mpox, while VACV L1 showed some specificity for vaccinated individuals (9). However, no single antigen appears to be able to distinguish vaccinated from infected individuals (10). Among multiplex immunoassays, the Meso Scale Discovery (MSD) Orthopoxvirus Panel 1 offers simultaneous quantitative analysis of five pairs of homologous MPXV and VACV antigens (not including MPXV B21R and MPXV A27), which have previously been associated with neutralizing antibody (NAb) responses and virus functions crucial for infectivity (11, 12) (Table S1).

The aim of this serological study was to evaluate different serological methods for their ability to differentiate between naturally acquired immunity to mpox- and vaccine-induced antibody responses. We compare the performance of serological gold-standard methods (MPXV/VACV NT and MPXV IF) using whole viruses as antigens, against an MSD-kit including 10 different orthopox virus antigens.

## MATERIALS AND METHODS

### Study population

A total of 148 male individuals regularly attending HIV Pre-Exposure Prophylaxis (PrEP)-related appointments at the sexual health clinic “Venhälsan” at Södersjukhuset, Stockholm, were included during November 2022. Individuals were classified into PCR-confirmed mpox-infected, MVA-BN-vaccinated, or unexposed to infection or vaccine according to timepoint of exposure (infection, vaccine dose 1, or vaccine dose 2) before study inclusion (Table 1). Twelve individuals classified as “unexposed” were excluded from the primary analysis and model establishment due to likely previous childhood smallpox-vaccination based on date of birth (13). Their average antibody responses were significantly (*P* < 0.05) higher than the rest of the unexposed population (Fig. S1). These individuals were later used to validate the model. Participation in the study was voluntary and participants signed a written form of consent before inclusion. Ethical permission for the study was granted through the Swedish Ethical Review Authority (Permit number: 2022-02235-01).

**TABLE 1:** Overview of the study population and classification of study participants^*f*^

Classification	*N*	% of total	Median time after exposure (days)
All	148		
Participants excluded from primary analysis^*a*^	12		
Participants included in primary analysis	136	100	
Age (in years)			
Mean	38.7		
SD	10.0		
Minimum	20.00		
25% Percentile	31.00		
Median	37.00		
75% Percentile	44.00		
Maximum	76.00		
Range	56.00		
Infected^*b*^^,^^*c*^	22	16.2	91
Vaccinated^*d*^			
Total	53	39.0	35
One dose	42	31.0	37
Two doses	11	8.1	25
Unexposed^*e*^	61	49.3	

^*a*^ Participants excluded due to likely previous smallbox vaccination based on date of birth (see Materials and Methods). This population is used for later model validation (Fig. 4).

^*b*^ *n* = 22/22 analyzed in MPXV- and vaccinia virus-specific NT. Due to lack of serum material *n* = 20/22 analyzed in MPXV-specific IF and *n* = 21/22 MSD assay.

^*c*^ PCR-confirmed MPXV infection ≥14 days prior to study inclusion.

^*d*^ One dose: Vaccinated with one dose JYNNEOS ≥14 days prior to study inclusion; two doses: Vaccinated with two doses JYNNEOS ≥ 14 days prior to study inclusion; booster vaccination administered at least 28 days after first dose.

^*e*^ Not fulfilling above criteria for vaccination/infection.

^*f*^ Empty cells in the "Median time after exposure (days)" column reflect that this value is applicable only to a limited number of parameters.

### Viruses and cell culture

A VACV and MPXV strain (22-05356 p2 220629, Clade 2b (14),) were used for microneutralization as well as immunofluorescence testing.

The viruses were propagated in confluent Vero cell cultures and titrated via end-point-dilution assay. Briefly, cells were infected in complete MEM at a multiplicity of infection (MOI) of 0.1 for 1 h. On day 4, with ≥50% of the culture-surface area consisting of visible plaques, the supernatant and cells were harvested and centrifuged at 2,000 *g* for 2 min. The cell pellets were resuspended in 500 µL medium and vortexed thoroughly. Cell suspensions were then lysed by freeze-thawing three times in a dry-ice ethanol bath and lysates were resuspended in the supernatant previously saved and centrifuged at 2,000 *g* for 5 min to remove the pellet.

For titration, Vero cells in 96-well plates were treated with virus solution in a dilution series of 10-1 to 106 with eight wells per dilution. After 5 days, the cells were analyzed for infection-induced cytopathic effect (CPE) with a light microscope. Wells that were positive for CPE were marked and the number of 50% tissue culture infectious doses (TCID50) per milliliter was calculated using the Spearman-Karber method.

### Microneutralization test

Vero cells were seeded in 96 well flat bottom plates at 20.000 cells/well 24 h before assay-start. Heat-inactivated (56°C, 30 min) patient serum was serially diluted in growth medium using a twofold dilution from 1:2.5 to 1:320. Sixty microliters of each dilution was then mixed with 60 µL of 2000 TCID50 MPXV or VACV (100 TCID50 per 96-well), resulting in a final serum dilution from 1:5 to 1:640. Both VACV and MPXV-serum mixtures were prepared in duplicates and incubated for 1 h at 37°C. After that, 100 µL of serum-virus mixture was added to Vero cells and incubated at 37°C and 5% CO_2_.

CPE was assessed after 5 days’ incubation. Assessment was done by optical microscopy, and neutralization was defined as <50% of the cell layer presenting with CPE and no neutralization if ≥50% of the cell layer presented with CPE. Final patient titers were calculated as the arithmetic mean of the highest neutralizing dilution titer of each duplicate per sample.

### Immunofluorescence assay

Infected cells were mixed with uninfected Vero cells. The cells were allowed to attach to cleaned 12-well slides and slides were with cold anhydrous acetone. Slides were stored at −70°C until used.

Sera were diluted in NaCl at twofold dilutions starting at 1:10. The diluted sera were incubated on the IFA-slides for 30 min at 37°C followed by a washing step. Alexa Fluor 488 AffiniPure Goat Anti-Human IgG (Jackson ImmunoResearch Laboratories, Inc., Baltimore, USA) was diluted in Evans-blue (Beijing Solarbio Scientific & Technology Co, Ltd.) in 1:200, and slides were incubated with the conjugates for 30 min at 37°C. Incubation was followed by a washing step and fixation with a glycerol/glycine-solution until reading with a Nikon Eclipse Ni fluorescens microscope (Nikon Inc., Japan).

### Multiplex serology assay for orthopox viruses

The V-PLEX Orthopoxvirus Panel 1 (IgG) Kit (Meso Scale Diagnostics, USA) (MSD Orthopox assay) was performed according to the manufacturer’s protocol. Multiple serum sample dilutions were initially tested to avoid saturation in positive samples; subsequent tests used a 1:2,000 dilution. Raw luminescent data were obtained with MesoScale QuickPlex SQ 120, and data were analyzed with MSD Discovery Workbench.

### Statistics

IF and NT titers were analyzed for sensitivity and specificity using Microsoft Excel. Assay data of the MSD Orthopox assay were analyzed with Discovery Workbench 4.0 (Meso Scale Diagnostics, USA). Statistical analysis, plotting, and normality testing were performed using GraphPad Prism 10 (GraphPad, USA).

Calculating optimal cut-offs for receiver operating characteristic (ROC) curves were performed with R (R Foundation for Statistical Computing, Vienna, Austria). Stratified repeated *k*-fold cross-validation (*k* = 10, 1000 repeats) was used to evaluate the robustness of the threshold selection process for each antibody response ratio as well as to assess the impact of the choice of threshold on the out-of sample predictive performance in discriminating vaccinated from naturally infected individuals.

For each repeat, the data were split into 10 stratified folds, i.e., ensuring that the proportion of vaccinated vs naturally infected individuals was maintained within each fold. Nine folds were used for training, while the remaining fold was reserved for testing. Within each set of training folds, an “optimal” classification threshold was identified based on the Youden Index of a receiver operating characteristic (ROC) curve. The “optimal” threshold was then used to calculate the out-of-sample sensitivity, specificity, and accuracy on the test fold.

The process was iterated across all folds within each repeat, and the entire procedure was repeated 1,000 times using different random stratified splits to ensure stability of estimates and to account for variation due to random fold assignment.

Out-of-sample sensitivity, specificity, and accuracy were summarized as the median of the distribution across 1,000 repeats (averaging across-folds within each repeat) with 95% CI calculated as the 2.5th and 97.5th percentiles of each metric distribution. In addition, the median and 95% percentile range of the optimal thresholds was calculated to provide an assessment of the robustness of the threshold selection process.

## RESULTS

### Antibody responses against whole virus in microneutralization or immunofluorescence assays cannot distinguish between previously infected and vaccinated individuals

**Fig 1:**
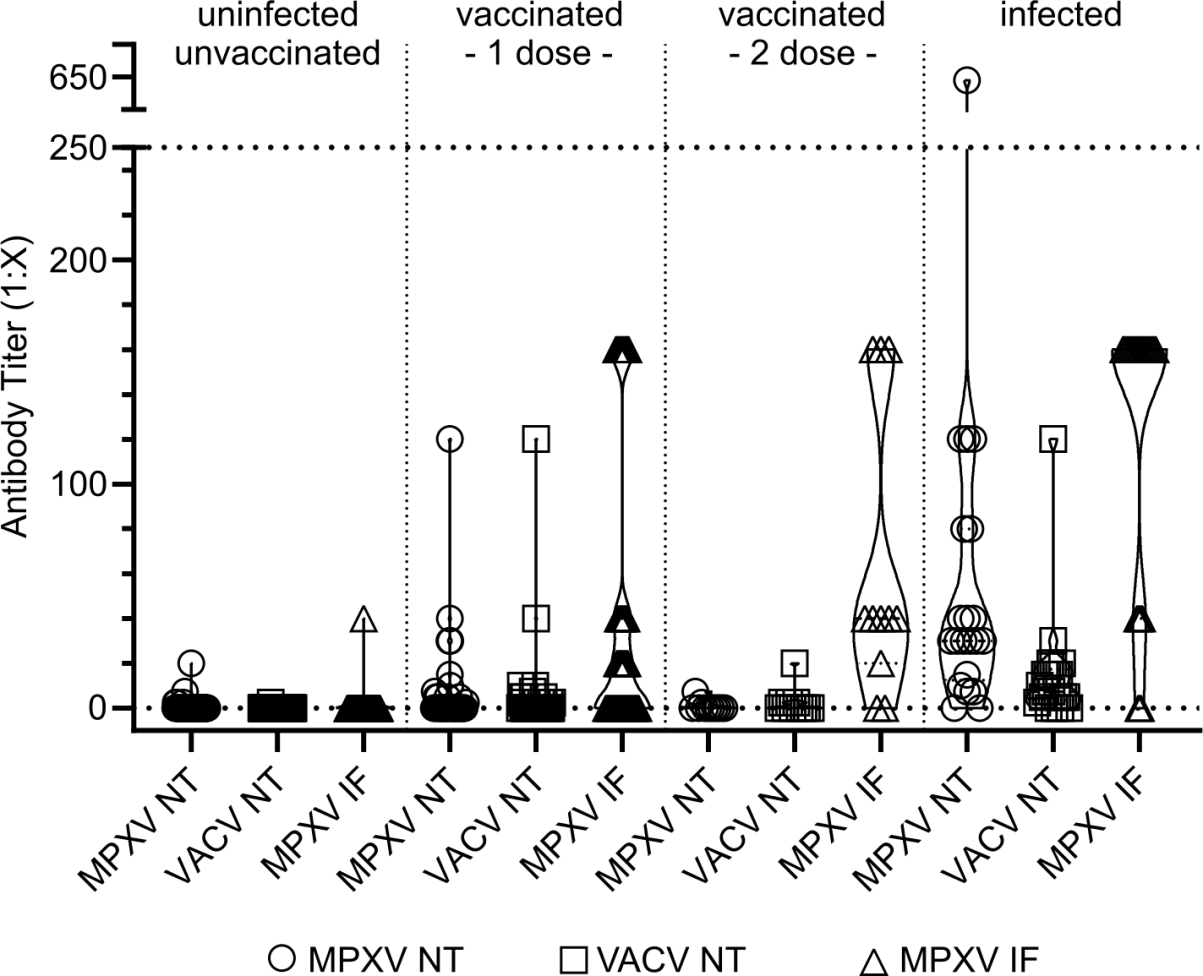
Antibody responses to mpox infection and vaccination using gold-standard serological methods. Neutralizing antibody titers determined by MPXV NT and VACV NT, respectively, as well as binding antibody titers determined by MPXV IF after mpox infection or vaccination. MPXV = monkeypoxvirus; VACV = vacciniavirus; NT = microneutralization test; IF = immunofluorescence.

To study the humoral immune response after mpox infection and vaccination with MVA-BN, respectively, NAbs against MPXV and VACV, as well as binding antibodies (BAbs) against MPXV were determined (Table S2). Out of the individuals with previous mpox infection, 90.9% were positive for NAbs against MPXV, whereas only 26.4% of the previously MVA-BN-vaccinated individuals were positive for NAbs. However, similar levels of BAb levels were detected after infection (90%) and two-dose-MVA-BN-vaccination (81.8%), respectively (Fig. 1; Table S2). The assay sensitivity was higher for MPXV-related antibody responses than for VACV (Table 2). However, the gold-standard methods were characterized by high cross-reactivity, impairing distinction between unexposed, vaccinated, and infected individuals with single thresholds (Table 2).

**TABLE 2:** Sensitivity and specificity of gold-standard methods to determine MPXV-infection/vaccination antibody-response^*a*^

	MPXV infected vs unexposed		Vaccinated vs unexposed		% of MVA-BN-vaccinated with positive test result^*b*^	% MPXV-infected with positive test result^*b*^
	Sensitivity%	Specificity%	Sensitivity%	Specificity%		
MPXV IF	90.0	98.4	NA^*c*^	NA	56.6	NA
MPXV NT	90.9	91.8	NA	NA	26.4	NA
VACV NT—total	NA	NA	32.1	98.4	NA	77.3
VACV NT—one dose	NA	NA	31.0	98.4	NA	NA
VACV NT—two dose	NA	NA	36.4	98.4	NA	NA

^*a*^ All assays are considered positive when dilution >0.

^*b*^ Single cut-off >0 is used.

^*c*^ NA, not applicable.

### IgG against single MPXV and VACV antigens distinguish unexposed from exposed individuals but cannot distinguish between infected and MVA-BN-vaccinated individuals

**Fig 2:**
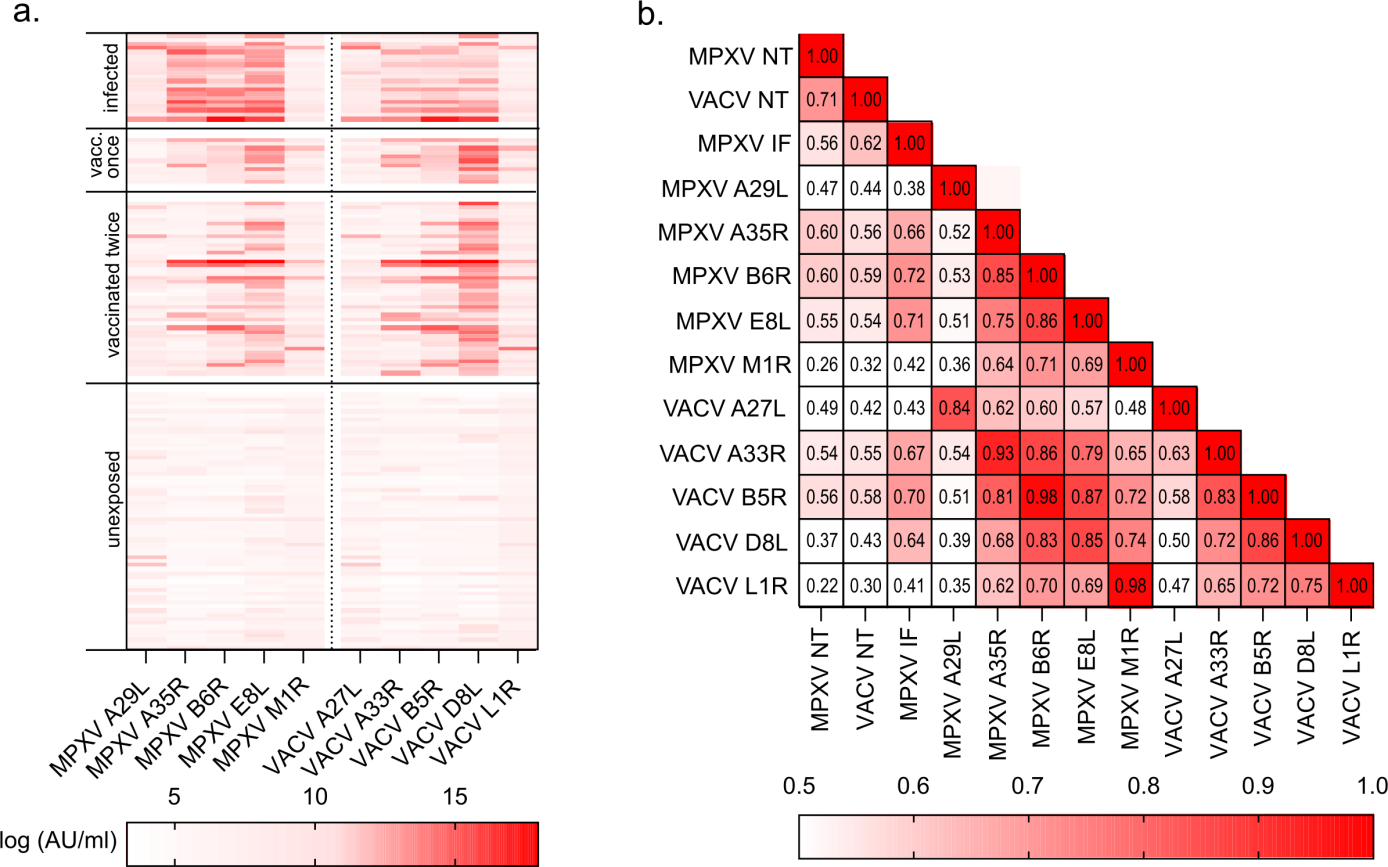
Induction of antibody responses against MPXV- and VACV-specific antigens after both infection and vaccination. (a) Heatmap of antibody responses to MPXV and VACV specific proteins, units: log (AU/mL). (b) Heatmap of Spearman correlation coefficients *r* between MPXV- and VACV-NT, MPXV IF, and antigens included in the V PLEX orthopoxvirus Panel 1 kit. AU = antibody unit concentration.

Next, antibody responses to five different MPXV antigens and their VACV homologs were measured using the MSD Orthopox assay. Both vaccination and infection significantly induced both MPXV- and VACV-specific IgG responses (*P* < 0,0001, Kruskal–Wallis followed by Dunn’s post hoc test with correction for multiple comparisons, Fig. 2A) compared to the unexposed study participants. Individual cut-off values for each antigen were established as mean concentration across the unexposed group + 2 standard deviations (Table S3) and sensitivity and specificity were calculated for all individual antigens both for infected/vaccinated combined, as well as separately (Table 3). All antigens used in the assay allowed for differentiation of the unexposed group from the exposed groups (specificity for unexposed > 90%, Table 3). However, high cross-reactivity between MPXV and VACV antigens (Table 3) prevented the differentiation of previously infected and MVA-BN-vaccinated individuals based on single antigen reactivity (Table 3).

**TABLE 3:** IgG assay sensitivities and specificities of MPXV-specific antigens included in the MSD V-Plex orthopoxvirus Panel 1^*a*^

	[MPXV infected + vaccinated] vs unexposed*		MPXV-infected vs unexposed*		MPXV-infected vs vaccinated**	
	Sensitivity%	Specificity%	Sensitivity%	Specificity%	Sensitivity%	Specificity%
MPXV A29L	12.2	96.7	23.8	96.7	14.3	96.2
MPXV A35R	55.4	93.4	95.2	93.4	14.3	98.1
MPXV B6R	77.0	95.1	95.2	95.1	4.8	98.1
MPXV E8L	79.7	93.4	95.2	93.4	0.0	98.1
MPXV M1R	29.7	96.7	23.8	96.7	4.8	98.1

			Vaccinated vs unexposed*		Vaccinated vs MPXV-infected***	
VACV A27L	13.5	96.7	11.3	96.7	0.0	90.5
VACV A33R	56.8	95.1	43.4	95.1	5.7	90.5
VACV B5R	82.4	93.4	77.4	93.4	1.9	95.2
VACV D8L	95.9	90.2	98.1	90.2	5.7	95.2
VACV L1R	36.5	96.7	41.5	96.7	9.4	95.2

^*a*^ Cut-off is calculated from the unexposed (*), vaccinated (**), or MPXV-infected (**) groups.

To study the relationship between the analyzed serological methods, Spearman Correlation Coefficients were calculated. For all samples, titers determined by gold-standard methods MPXV NT correlated well with VACV NT and IF titers (Fig. 2b). Notably, IgG responses against specific MPXV-VACV-antigen pairs correlated (*r* > 0.85), raising the question if the use of both VACV- and MPXV-antigens in the same assay can be exploited for mpox infection/vaccination status determination.

### The response ratios to homologous antigen pairs A35R-A33R, B6R-B5R, E8L-D8L separate infected and MVA-BN-vaccinated individuals

**Fig 3:**
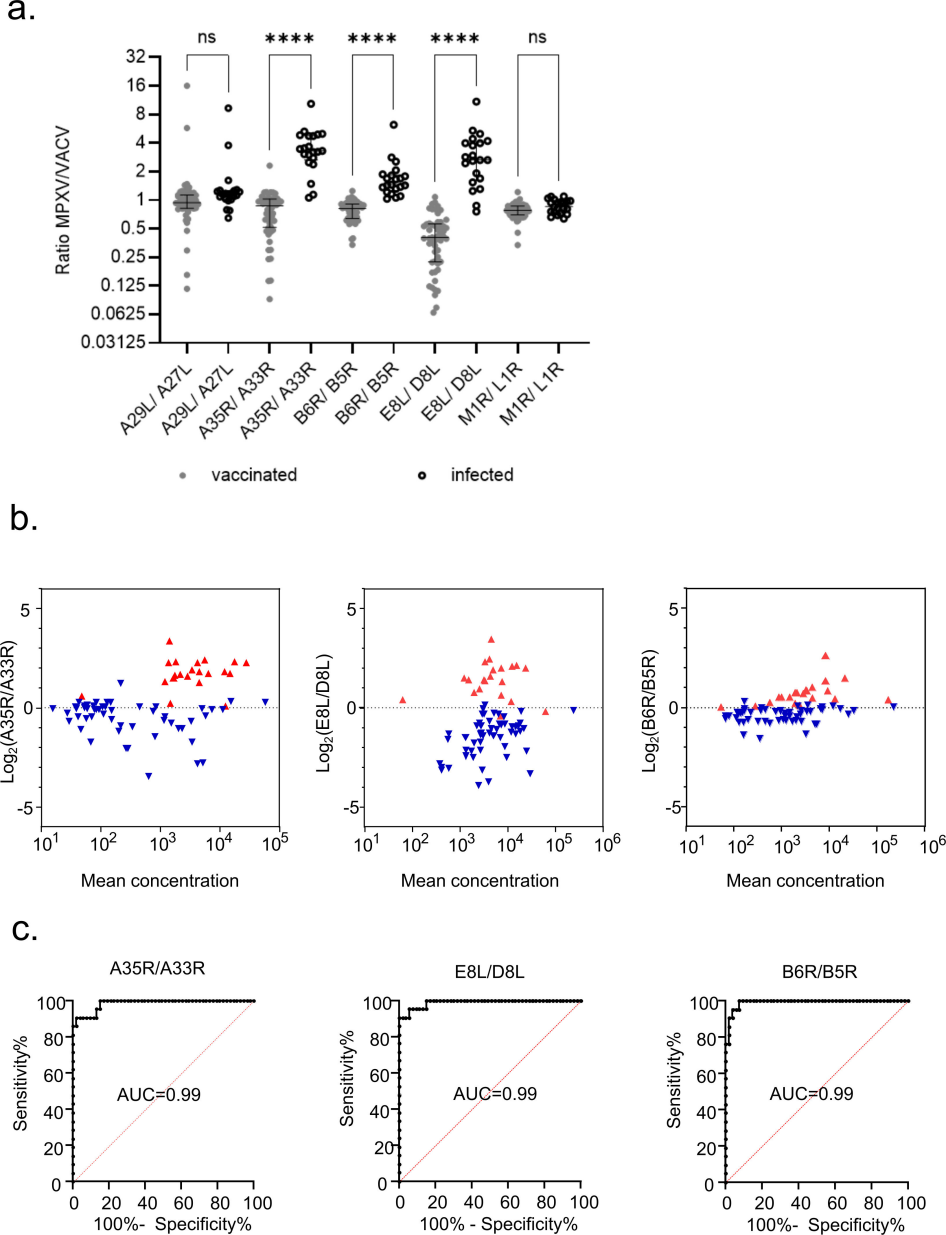
MPXV/VACV antibody response ratios differentiate between mpox-infected and vaccinated samples. (a) A29L/A27L, A35R/A33R, B6R/B5R, E8L/D8L, and M1R/L1R ratios were compared between vaccinated and MPXV-infected samples. Kruskal-Wallis test; ****P < 0.0001; ns, non-significant. (b) MA (log ratio vs mean antibody concentration across the two homologous MPXV and VACV antigens)-plots of A35R/A33R- (left panel), E8L/D8L- (middle panel), and B6R/B5R- (right panel) ratios. Blue triangles: vaccinated samples; Red triangles: MPXV-infected samples. (c) Receiver operating characteristic (ROC) curves of A35R/A33R (left panel), E8L/D8L (middle panel), and B6R/B5R (right panel) ratios depict the separability of vaccinated and MPXV-infected samples based on MPXV/VACV antibody response ratios. AUC, area under the ROC curve. MPXV-infected: *n* = 21; vaccinated: *n* = 53.

The relationship between the antigen homologs was studied in more depth. Analysis of the response-ratios revealed significantly higher levels of differential antibody responses against MPXV-specific surface proteins A35R, B6R, and E8L compared to their VACV homologs (*P* < 0.0001) after mpox infection compared to vaccination (Fig. 3a). MA-plots of MPXV/VACV-IgG-concentration ratios of corresponding IgG MPXV- and VACV-homologs from previously infected and vaccinated study participants, indicated that ratios of the homologous antigen pairs A35R-A33R, B6R-B5R, E8L-D8L showed higher separation between the study groups compared to the antigen pairs without significantly differential responses (Fig. 3b; Fig. S2a).

To quantify the reliability of this separation and the diagnostic utility of the ratio calculation, single antigen-response-ratios of all five homologous pairs were plotted in Receiver Operator Characteristic (ROC) curves (Fig. 3c; Fig. S2b). High values (0.99) of areas under the ROC curves of the three A35R/A33R-, B6R/B5R-, and E8L/D8L-concentration ratios indicate a good ability of the ratios to discriminate between infected and vaccinated study participants (Fig. 3c).

An optimal classification threshold to differentiate MPXV-infected from vaccinated was identified based on the Youden Index of a ROC curve. Stratified repeated *k*-fold cross-validation was used to evaluate the robustness of the threshold selection process and the out-of-sample sensitivity, specificity, and accuracy.

Individual sensitivity and specificity calculations for the three antigen concentration ratios—A35R/A33R, E8L/D8L, and B6R/B5R —demonstrated high sensitivity (86.0%, 86.0%, and 91.0%, respectively) and high specificity (94.2%, 94.5%, and >93.3%, respectively). Prediction accuracy reached up to 93.2% for B6R/B5R, 93.1% for E8L/D8L, and 91.9% for A35R/A33R (Table 4).

**TABLE 4:** Optimal MPXV/VACV ratio cut-off values and prediction accuracy^*a*^^,^^*b*^

			Sensitivity		Specificity			
	Optimal cut-off	95% CI	%	95% CI	%	95% CI	% prediction accuracy	95% CI
A35R/A33L	1.50	1.32–1.69	86.0	80.0–91.0	94.2	88.2–100	91.9	86.7–96.0
E8L/D8L	1.10	1.00–1.22	86.0	80.0–01.2	94.5	88.6–98.2	93.1	87.8–95.4
B6R/B5R	1.05	1.04–1.07	91.0	85.0–96.0	93.3	92.2–96.2	93.2	90.5–94.8

^*a*^ Cross-validated median optimal thresholds and out-of-sample performance metrics with 95% CI based on stratified repeated *k*-fold cross-validation using 10 folds and 1,000 repeats. CI, confidence interval.

^*b*^ Optimal ratio cut-offs were calculated with the Youden Index and sensitivity and specificity were estimated between MPXV-infected and vaccinated groups for each antigen pair.

### Differentiation of MPXV-infected from MVA-BN-vaccinated and unexposed cohorts with multiplex serological assay

**Fig 4:**
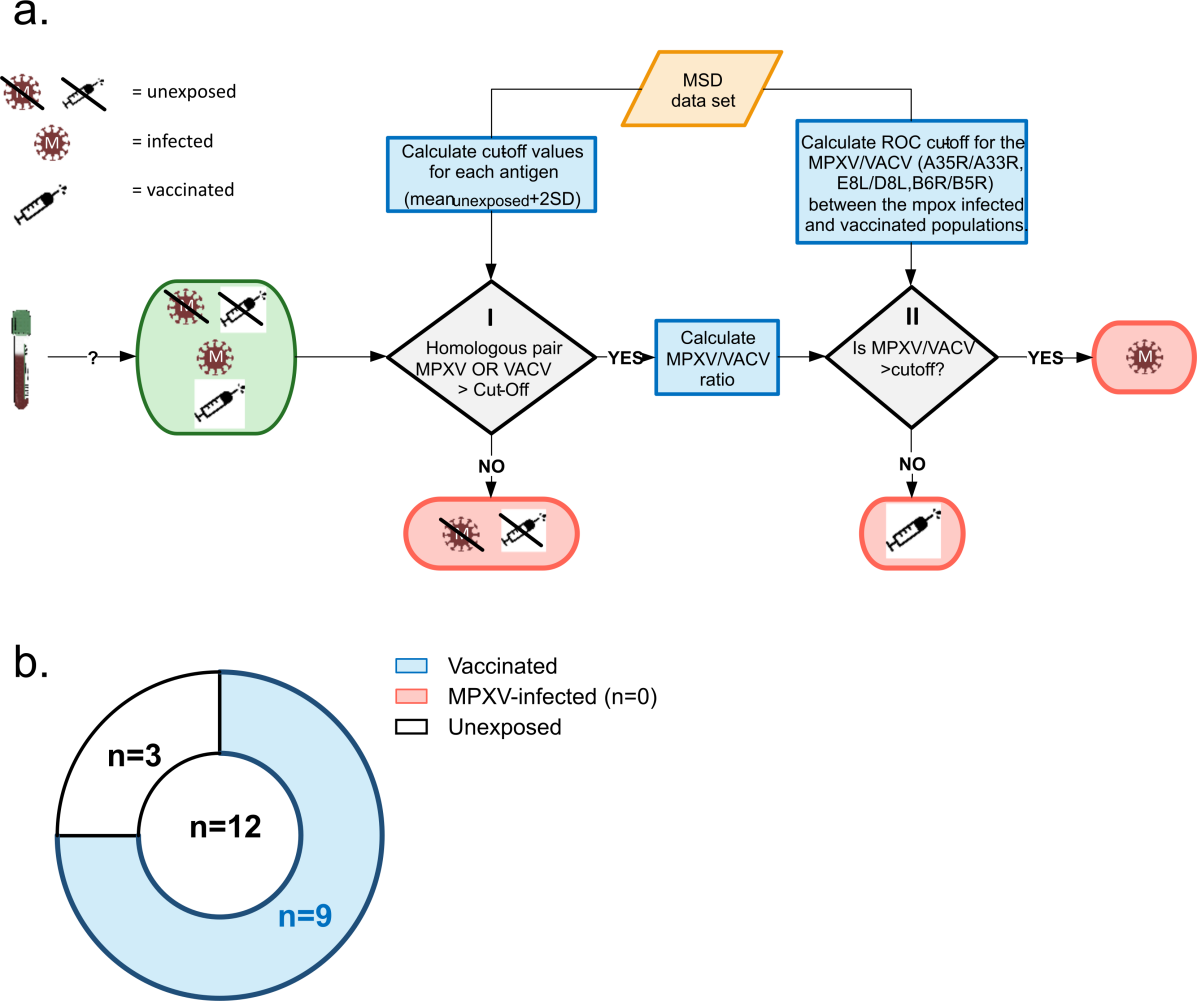
Differentiation of MPXV-infected from vaccinated and unexposed cohorts with multiplex serological assay. (**a**) Analysis workflow of MPXV serology based on homologous MPXV- and VACV-specific antigen-pairs. Each antigen threshold from unexposed (Table S3) was applied on all included study participants, and a sample was considered positive if either the homologous MPXV or VACV signals were >cut off. MPXV/VACV ratio in positive samples was tested with optimal ROC thresholds (Table 4) for infection/ vaccination status. (**b**) Prediction of the combination model on the excluded >1977 born individuals.

Summarizing all findings, a model for MPXV serology testing was established, based on the specific, homologous MPXV- and VACV-antigen-pairs A35R/A33R, B6R/B5R, and E8L/D8L (Fig. 4a).

Application of the model on all included study participants revealed that each antigen pair had its individual strength and weakness in predicting exposure status (Table 5; Table S4). Utilizing all three antigen pairs within a combined model demonstrated a notable strength in accurately discriminating between unexposed and mpox-infected individuals, yielding an error rate below 5%. However, a fifth of the samples from MVA-BN-vaccinated participants were classified as negative, indicating a trade-off in sensitivity favoring the correct diagnosis of mpox-infected individuals. Interestingly, when applying the combined model separately to individuals vaccinated once or twice, prediction accuracy increased from 73.81% in the one-dose group to 100% in the two-dose group. This improvement was also evident when examining individual antigen pairs: A35R/A33R achieved 40.48% and 54.54% accuracy in the one- and two-dose groups, respectively; E8L/D8L reached 97.62% and 100%; and B6R/B5R achieved 69.05% and 90.91% (Table S5).

**TABLE 5:** Predictive performance of the analysis model with the A35R/A33R, E8L/D8L, and B6R/B5R antigen-pairs when applied to the whole data set^*a*^

	% Overall prediction accuracy	% Prediction accuracy of unexposed	% Prediction accuracy of MPXV-infected	% Prediction accuracy of vaccinated
A35R/A33R	71.85	91.80	85.71	43.40
E8L/D8L	90.37	85.25	85.71	98.11
B6R/B5R	85.93	93.44	95.24	73.58
Combined^*b*^	88.89	96.72	90.48	79.25

^*a*^ Unexposed: *n* = 61; MPXV-infected: *n* = 21; vaccinated: *n* = 53.

^*b*^ Combination values were calculated from all three ratios by choosing MPXV-infected, vaccinated, or unexposed classification according to the highest (at least 2 out of 3) agreement between the A35R/A33R, E8L/D8L, and B6R/B5R predicted classifications.

In a second validation approach, the combination model was applied to the individuals excluded from primary analysis and model establishment due to likely previous smallpox-vaccination based on date of birth. 75% of samples were classified as smallpox-vaccinated and 25% unexposed (Fig. 4b), in agreement with the predictive performance (Table 5) estimate (when all persons born before 1977 assumed vaccinated).

## DISCUSSION

The study compared gold-standard serological diagnostic methods using whole viruses as antigens to a new approach, where the MSD Orthopox assay is combined with an innovative analytical approach. This method uses ratios of several viral antigens of importance to immune responses after mpox infection and vaccination, respectively, to assess their ability to differentiate between naturally acquired immunity to mpox and MVA-BN-vaccine-induced antibody responses. Our analyses revealed that the MSD serology assay is characterized by high sensitivity for the detection of previously mpox-infected individuals. Moreover, by using an analysis model leveraging response ratios to various viral antigens, the assay differentiates well MPXV-infected from MVA-BN vaccinated and unexposed individuals. Similar to Hunt et al. (10), we found that no single antigen used in the MSD Orthopox assay can distinguish vaccinated from infected individuals. Using multiplex orthopox serological assays, Otter (9), Hicks (15), and Jones (16) showed that the binding between MPXV and VACV homologous antigens varies depending on whether individuals were vaccinated with VACV or infected with MPXV. We built upon this concept and defined cutoffs in the concentration ratios of homologous VACV and MPXV antigens—A35R/A33R, B6R/B5R, and E8L/D8L. This enabled highly accurate discrimination between infected and vaccinated individuals using the MSD Orthopox panel assay.

Notably, recent work supports this concept, demonstrating that combining an MPXV-specific antibody (A27L), targeting an MPXV region not present in the MVA-BN-Vaccine, with a homologous antigen ratio (B6R/B5R) achieved high discriminatory power between post infection and post vaccination immune responses (17). This elegant approach highlights the strength of ratio-based serological discrimination and reinforces the utility of our findings for scalable and accurate population-level surveillance.

We combined classifications from the A35R/A33R, B6R/B5R, and E8L/D8L antigens. A particular strength of the combination model is that it increases the specificity for orthopox by providing better prediction accuracy for unexposed individuals than any of the individual antigens alone. The model is highly sensitive in recognizing samples from mpox-infected individuals but shows lower sensitivity in identifying MVA-BN-vaccinated individuals. Notably, the prediction of VACV seropositivity using this approach fails only in individuals who received a single dose of the vaccine, potentially also due to low seroconversion rates after one-dose vaccination (18).

Moreover, while the analysis approach is transferrable and applicable in many settings, the method for determining the optimal cut-offs might need to be adjusted since different clinical settings and applications require tailored balances between sensitivity and specificity (19). The orthopox positivity threshold was initially determined using the mean + 2 SD of unexposed samples (*n* = 61), targeting 97.7% specificity. To validate this approach, ROC curve analysis was also performed using both unexposed and MPXV-infected or vaccinated samples (depending on the antigen in question). The ROC-derived thresholds at approximately 97.7% specificity were comparable to the mean + 2 SD thresholds, confirming the validity of our initial threshold determination method (Table S6).

Moreover, the patient population and estimated seroprevalence need to be taken into account. Our “unexposed” group, drawn from a high-risk population, allowed for conservative MSD Orthopox cut-off estimates representative of the local MSM population. However, these cut-offs are likely too low for use in African populations, where assay backgrounds are presumed higher despite limited seroprevalence data (20).

A particular strength of this study is the comprehensive analysis of immune responses toward orthopoxviruses. Comparing the performance of a multiplex ELISA for measuring BAbs against well-established methods like NT or IF provides valuable information for the development of cost-effective methods to study mpox serology at low biosafety levels. While the standard methods MPXV NT and IF demonstrated high sensitivity for the detection of MPXV-specific antibodies after infection, the usage of whole virus in the assays comes with challenges for differentiation of infection/vaccination status, as expected. Moreover, the high correlation between the MPXV and VACV NT assays stresses that there is no benefit in running both assays since they are interchangeable.

While NAb have been used as a correlate of protection for many other infections, waning NAb responses after vaccination (21) and some re-infection rates (22) have rendered it challenging to identify a correlate of protection for mpox. Enabling a more in-depth look at single-antigen responses, while at the same time, benchmarking against gold-standard methods allows for a more comprehensive analysis of antibody responses and can contribute to establishing a correlate of protection in future studies. The cross-sectional study design did not allow any analyses of longitudinal antibody response, but immune responses to specific VACV- and MPXV-specific antigens over time would be very interesting. Taking into account recent clinical findings about waning Nab responses after MVA-BN vaccination (23), better understanding of the quality, durability, and cross-reactivity of long-term immune responses—both vaccination-induced and infection-induced—is needed to guide future vaccine strategies.

In summary, our study provides a uniquely broad perspective on orthopox serology by contrasting the gold standard NT and IF methods with the novel MSD multiplex assay and by providing an analysis model of multiplex data to separate previously mpox-infected, MVA-BN-vaccinated, and unexposed individuals.

The findings from this study could be directly transferred to the development of affordable, high-throughput serological methods to address pressing questions like mpox seroprevalence, where an assay that can accurately distinguish natural infection from MVA-BN vaccination is key methodology for reliable disease surveillance.

## SUPPLEMENTAL MATERIAL

Supplemental Material spectrum.00182-25-s0001.docx Graphical abstract, Fig. S1 and S2, and Tables S1 to S6.

ASM does not own the copyrights to Supplemental Material that may be linked to, or accessed through, an article. The authors have granted ASM a non-exclusive, world-wide license to publish the Supplemental Material files. Please contact the corresponding author directly for reuse.
