# Supplementary material for: Serological differentiation between naturally acquired mpox and MVA-BN-vaccine induced antibody responses using ratios of MPXV and VACV antigen pairs in the MSD immunoassay
Source: Microbiol Spectr. 2025 Aug 8;13(9):e00182-25. doi: 10.1128/spectrum.00182-25 (PMC12403559; doi:10.1128/spectrum.00182-25)
Supplement: Supplemental Material — Graphical abstract, Fig. S1 and S2, and Tables S1 to S6. [file spectrum.00182-25-s0001.docx]

# Differentiation of naturally acquired immunity to mpox from vaccine induced antibody responses - evaluation of neutralization assay, IF and Mesoscale

## Supplementary material

## Graphical Abstract


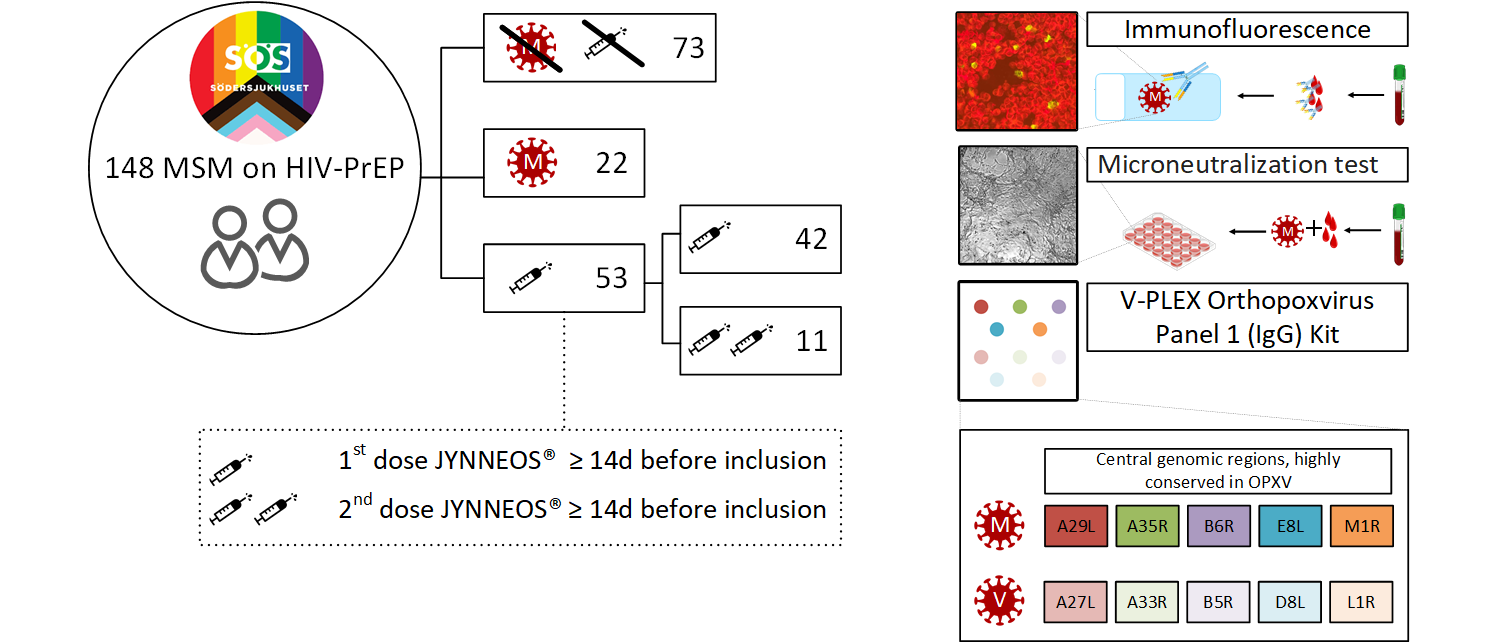

**Supplementary Figure 1.** a) Mean(+SEM) titers of MPXV NT, VACV NT and MPXV IF in unexposed study population born before or after 1977 b) Concentrations of specific MPXV and VACV IgG antibodies measured with the MSD Orthopoxvirus Panel1. DOB: date of birth.

**Supplementary Table 1.** Proteins included in the V-PLEX Orthopoxvirus Panel 1 (IgG) Kit

| **MPXV** | **VACV** | **Orthopoxvirus gene nomenclature** | **Protein function (1)** |  |
| --- | --- | --- | --- | --- |
| A29L | A27L | OPG154 | vesicle membrane protein found on the surface of IMV particles, MV wrapping  EEV membrane membrane phosphoglycoprotein, actin tail formation  EEV envelope membrane glycoprotein, WV formation, target of protective antibodies  IMV membrane protein, inactive carbonic anhydrase homolog, GAG-binding  IMV surface membrane protein, EFC component, neutralizing antibody target | |
| A35R | A33R | OPG161 |  |  |
| B6R | B5R | OPG190 |  |  |
| E8L | D8L | OPG120 |  |  |
| M1R | L1R | OPG095 |  |  |

**Supplementary Table 2:** Antibody responses analysed by gold-standard methods

| Method | Exposure status | Total tested | Positives | % |
| --- | --- | --- | --- | --- |
| **MPXV- NAb (any NT-Titer)** |  |  |  |  |
|  | after infection | 22 | 20 | 90.9 |
|  | 1-dose vaccination | 42 | 12 | 28.6 |
|  | 2-dose vaccination | 11 | 2 | 18.2 |
|  | unexposed | 61** | 5 | 8.2 |
|  |  |  |  |  |
| **MPXV – BAb (any IF-titer)** |  |  |  |  |
|  | after infection | 20* | 18 | 90.0 |
|  | 1-dose vaccination | 42 | 21 | 50.0 |
|  | 2-dose vaccination | 11 | 9 | 81.8 |
|  | unexposed | 61** | 1 | 1.6 |
|  |  |  |  |  |
| **VACV- NAb (any NT-Titer)** |  |  |  |  |
|  | after infection | 22 | 17 | 77.3 |
|  | 1-dose vaccination | 42 | 13 | 31.0 |
|  | 2-dose vaccination | 11 | 4 | 36.4 |
|  | unexposed | 61** | 1 | 1.6 |
|  |  |  |  |  |
| * not enough serum for tests of two individuals | | | | |
| ** 12 individuals born before 1977 excluded from primary analysis | | | | |

**Supplementary Table 3.** MSD V-Plex orthopoxvirus Panel 1 assay individual concentration (AU/ml) cut-off values were calculated for each antigen using the unexposed group.

|  |  | Average (Avg) | Standard Deviation (SD) | Cut-off  (Avg + 2SD) |
| --- | --- | --- | --- | --- |
| MPXV | MPXV A29L | 226.4 | 568.0 | 1362.4 |
|  | MPXV A35R | 57.2 | 63.8 | 184.8 |
|  | MPXV B6R | 62.8 | 64.4 | 191.7 |
|  | MPXV E8L | 165.7 | 262.7 | 691.2 |
|  | MPXV M1R | 116.2 | 149.6 | 415.5 |
| VACV | VACV A27L | 170.9 | 395.4 | 961.8 |
|  | VACV A33R | 72.9 | 120.3 | 313.4 |
|  | VACV B5R | 66.5 | 63.6 | 193.8 |
|  | VACV D8L | 157.3 | 288.5 | 734.3 |
|  | VACV L1R | 134.7 | 143.1 | 420.8 |

**Supplementary Table 4.** Confusion matrices of predictive performance of the analysis model with the A35R/A33R, E8L/D8L, and B6R/B5R antigen-pairs.

|  |  | **True exposure status** | | | | | | | | | | | |
| --- | --- | --- | --- | --- | --- | --- | --- | --- | --- | --- | --- | --- | --- |
| **Predicted exposure status** |  | **U** | **M** | **V** | **U** | **M** | **V** | **U** | **M** | **V** | **U** | **M** | **V** |
|  | **U** | 56 | 1 | 29 | 52 | 1 | 1 | 57 | 1 | 12 | 59 | 1 | 11 |
|  | **M** | 1 | 18 | 1 | 3 | 18 | 0 | 1 | 20 | 2 | 0 | 19 | 0 |
|  | **V** | 4 | 2 | 23 | 6 | 2 | 52 | 3 | 0 | 39 | 2 | 1 | 42 |
|  |  | **A35R/A33L** | | | **E8L/D8L** | | | **B6R/B5R** | | | **Combined** | | |

U: Unexposed; M: MPXV-infected; V: vaccinated

0

20

40

60

80

100

0

20

40

60

80

100

A29L/A27L

100% - Specificity%

0

20

40

60

80

100

0

20

40

60

80

100

M1R/L1R

100% - Specificity%

Sensistivity%

Sensistivity%

AUC=0.69

AUC=0.66

-5

0

5

-5

0

5

Mean concentration

10

1

10

2

10

3

10

4

10

5

Mean concentration

10

1

10

2

10

3

10

4

10

5

Log_2_(A29L/A27L)

Log_2_(M1R/L1R)

a.

b.

**Supplementary Figure 2.** **MPXV/VACV antibody response ratios of responses to A29L/A27L- and M1R/L1R-antigens do not differentiate between mpox infected and vaccinated samples** a) MA-plots of A29L/A27L- (left panel), and M1R/L1R- (right panel) ratios. Blue triangles: vaccinated samples; Red triangles: MPXV-infected samples

b) Receiver operating characteristic (ROC) curves of A29L/A27L- (left panel), and M1R/L1R- (right panel) ratios. AUC: area under the ROC curve.

**Supplementary Table 5 (with 2SD orthopox seropositivity cutoff).** Predictive performance of the analysis model with the A35R/A33R, E8L/D8L, and B6R/B5R antigen-pairs when applied to the split vaccinated dataset*.

|  | **% Prediction accuracy of**  **total vaccinated** | **% Prediction accuracy of 1 dose vaccinated** | **% Prediction accuracy of 2 dose vaccinated** |  |
| --- | --- | --- | --- | --- |
|  |  |  |  |  |
| **A35R/A33R** | 43.40 | 40.48 | 54.54 |  |
| **E8L/D8L** | 98.11 | 97.62 | 100 |  |
| **B6R/B5R** | 73.58 | 69.05 | 90.91 |  |
| **Combined**** | 79.25 | 73.81 | 100 |  |

*One dose: n=42; two doses: n=11; total: n=53

**Supplementary Table 6** Orthopox seropositivity cut-offs calculted with ROC analysis. Given

the discrete nature of the ROC curve, threshold values at 96.7% and 98.4% specificity were selected as the nearest lower and upper bounds, respectively, to the target 97.7% specificity.

|  |  | **Threshold** | | |  |  |
| --- | --- | --- | --- | --- | --- | --- |
|  |  | **Original mean+2SD** | **ROC _96,7_ (∆threshold*)** | **ROC _98,4_ (∆threshold*)** | **AUC** | **Senstivity%** |
| **MPXV-infected vs. Unexposed** | **MPXV A29L** | 1362.4 | 2300 (1.65) | 3102 (3.06) | 0.883 | 14.29 |
|  | **MPXV A35R** | 184.8 | 195.5 (0.17) | 299.7 (1.80) | 0.991 | 95.24 |
|  | **MPXV B6R** | 191.7 | 256.6 (1.01) | 281.1 (1.39) | 0.984 | 95.24 |
|  | **MPXV E8L** | 691.2 | 886.0 (0.74) | 1283 (2.25) | 0.980 | 95.24 |
|  | **MPXV M1R** | 415.5 | 572.0 (1.05) | 825.1 (2.74) | 0.798 | 9.52 |
| **Vaccinated vs. Unexposed** | **VACV A27L** | 961.8 | 1635 (1.70) | 1749 (1.99) | 0.699 | 5.66 |
|  | **VACV A33R** | 313.4 | 472.3 (1.32) | 627.2 (2.61) | 0.862 | 35.85 |
|  | **VACV B5R** | 193.8 | 251.9 (0.91) | 301.6 (1.69) | 0.9681 | 69.81 |
|  | **VACV D8L** | 734.3 | 1124 (1.35) | 1248 (1.78) | 0.992 | 88.68 |
|  | **VACV L1R** | 420.8 | 727.5 (2.14) | 745.0 (2.26) | 0.845 | 22.64 |

1. Senkevich TG. Yutin N. Wolf YI. Koonin EV. Moss B. Ancient Gene Capture and Recent Gene Loss Shape the Evolution of Orthopoxvirus-Host Interaction Genes. mBio. 2021;12(4):e0149521.
